# A Risk Stratification Model for Cardiovascular Complications during the 3-Month Period after Major Elective Vascular Surgery

**DOI:** 10.1155/2018/4381527

**Published:** 2018-09-09

**Authors:** Mladjan Golubovic, Dragana Stanojevic, Milan Lazarevic, Velimir Peric, Tomislav Kostic, Miodrag Djordjevic, Sasa Zivic, Dragan J. Milic

**Affiliations:** ^1^Clinic for Anesthesia and Intensive Care, Clinical Center Nis, 18000 Nis, Serbia; ^2^Clinic for Cardiovascular Diseases, Clinical Center Nis, 18000 Nis, Serbia; ^3^Clinic for Cardiothoracic and Transplantation Surgery, Clinical Center Nis, 18000 Nis, Serbia; ^4^University of Nis, Medical School Nis, 18000 Nis, Serbia; ^5^Clinic for General Surgery, Clinical Center Nis, 18000 Nis, Serbia

## Abstract

**Introduction:**

The Revised Cardiac Risk Index (RCRI) is an extensively used simple risk stratification tool advocated by the European Society of Cardiology and European Society of Anesthesiology (ESC/ESA).

**Purpose:**

The aim of this study was to find the best model for predicting 3-month cardiovascular complications in elective major vascular surgical patients using preoperative clinical assessment, calculation of the RCRI and Vascular Physiological and Operative Severity Score for the enumeration of mortality and morbidity (V-POSSUM) scores, and the preoperative levels of N-terminal brain natriuretic peptide (NT pro-BNP), high-sensitivity troponin I (hs TnI), and high-sensitivity C-reactive protein (hs CRP).

**Materials and Methods:**

We included 122 participants in a prospective, single-center, observational study. The levels of NT pro-BNP, hs CRP, and hs TnI were measured 48 hours prior to surgery. During the perioperative period and 90 days after surgery the following adverse cardiac events were recorded: myocardial infarction, arrhythmias, pulmonary edema, acute decompensated heart failure, and cardiac arrest.

**Results:**

During the first 3 months after surgery 29 participants (23.8%) had 50 cardiac complications. There was a statistically significant difference in the RCRI score between participants with and without cardiac complications. ROC analysis showed that a combination of RCRI with hs TnI has good discriminatory power (AUC 0.909, p<0,001). By adding NT pro-BNP concentrations to the RCRI+hs TnI+V-POSSSUM combination we obtained the model with the best predictive power for 3-month cardiac complications (AUC 0.963, p<0,001).

**Conclusion:**

We need to improve preoperative risk assessment in participants scheduled for major vascular surgery by combining their clinical scores with biomarkers. Therefore, it is possible to identify patients at risk of cardiovascular complications who need adequate preoperative diagnosis and treatment.

## 1. Introduction

Patients who are admitted for major noncardiac surgery should have risk stratification carried out according to the new guidelines, in addition to a standard physical examination and laboratory analyses [1, 2]. The Revised Cardiac Risk Index (RCRI) is a simple to use risk stratification method proposed by the ESC/ESA for all surgical patients [2–4].

The leading causes of death and morbidity in adults with major vascular surgical interventions are acute myocardial infarction, cardiac arrhythmias, and acute heart failure [5–8].

Surgical interventions on major arterial vessels are in the group of high-risk noncardiac surgical procedures for early mortality (above 5%) [9]. Meta-analyses showed that measuring brain natriuretic peptide (BNP) or N-terminal brain natriuretic peptide (NT pro-BNP) significantly increases the predictive power of RCRI, when they are used in combination with each other, for patients who are going to have vascular surgery [10]. Both normal and increased concentrations of these biomarkers carry information on postoperative risk [11]. Also, preoperative concentrations of high-sensitivity troponin I (hs TnI) carry prognostic information. Increased hs TnI preoperatively is associated with a higher risk of developing postoperative acute myocardial infarction (MI). Therefore, hs TnI also increases the predictive value of RCRI [12, 13]. High-sensitivity CRP (hs CRP) and NT pro-BNP measured preoperatively are good prognostic markers, in addition to the classically determined risk factors measured in patients planned for major vascular, noncardiac surgical interventions, according to Goei et al. [14].

Using The Vascular Physiological and Operative Severity Score to enumerate the mortality and morbidity (V-POSSUM) score, we can assess the risk of early mortality with high accuracy in patients scheduled for an aneurysm operation in vascular surgery [15, 16].

The aim of this research was to investigate whether using preoperatively determined biomarkers in different combinations with a patient's clinical scores can identify patients at high risk of cardiovascular complications during a 3-month period after elective vascular surgery.

## 2. Material and Methods

The Ethical Committee of the Medical Faculty at the University of Nis approved the research, which was conducted at the Clinic for cardiovascular and transplantation surgery, Clinical Center Nis, Serbia, during 2017. The primary inclusion criterion was a scheduled major open elective vascular surgery (abdominal aortic aneurysm surgery, infrainguinal arterial reconstruction, or carotid endarterectomy with general anesthesia) for patients older than 21 years. All participants (122) gave written informed consent for participation in this study before enrollment.

The exclusion criteria were unstable coronary artery disease and decompensated heart failure.

A routine clinical examination was carried out for all participants that included taking a detailed medical history, a physical examination, standard laboratory analyses, a 12-lead chest electrocardiogram, and a chest X-ray. With these data we used online software calculators to assess the risk scores. Using the site www.mdcalc.com/revised-cardiac-risk-index-pre-operative-risk we calculated the RCRI score for the development of cardiovascular complications. The V-POSSUM score was calculated using the software calculator: http://www.riskprediction.org.uk/vasc-index.php. Anesthetic management, perioperative care, and intensive care unit referral were at the discretion of the attending physicians.

The primary endpoints during the 90 days of follow-up were adverse cardiac events: acute myocardial infarction, cardiac arrhythmias, pulmonary edema, acutely decompensated heart failure, and cardiac arrest. We obtained information of any adverse events from scheduled controls and physical examination of participants and in the case of death outcome from analysis of medical records and death certificates.

Blood samples were taken within 48 hours prior to surgery from the antecubital vein and stored in serum vacutainer tubes without additives. NT pro-BNP (pg/ml) and hs TnI (ng/ml) were measured in the whole blood specimens using chemiluminescence enzyme immunoassay technology (CLEIA) and Magtration^®^ technology on a PATHFAST Immunoanalyser (Mitsubishi Chemical Europe GmbH, Düsseldorf, Germany). After centrifugation, the serum was separated and frozen at −80°C until analysis. Hs CRP (mg/L) and CK-MB (U/L) were detected in serum on a Beckman Coulter AU 680 analyzer (Beckman Coulter Inc., Brea, USA); the method is based on immunoturbidimetry.

### 2.1. Statistical Analysis

As a type of descriptive statistics, the data were presented in the form of their arithmetic mean and standard deviation and in the form of absolute or relative numbers. The normality of the data was tested using the Kolmogorov-Smirnov test. When comparing two data groups, if the normal distribution was satisfied, a T-test was used; otherwise, Mann–Whitney's U test was performed. To compare the attributes, a Chi-square test, or Fisher's exact probability test, was used. ROC analysis was used to evaluate the model's discrimination. In constructing a ROC curve for multiple variables from logistic regression analysis, a single variable is formed based on the probability of several individual variables. A comparison of multiple ROC curves was carried out using the DeLong test. Statistical significance was determined for a p value of less than 0.05. Net reclassification improvement (NRI) and integrated discrimination improvement (IDI) were calculated for different combinations of markers. All analyses were performed using the R package pROC and PredictABEL [17].

## 3. Results

During the first 3 months, 29 (23.8%) participants had 50 cardiovascular complications. During the follow-up period, 16 (55.2%) of these participants had one cardiovascular complication, eight participants (27.6%) had two complications, three participants had three complications (10.3%), one participant (3.4%) had four, and one participant had five complications (Table 1). One participant died due to acute MI. There were no participants with pulmonary embolism.

The occurrence of cardiovascular complications during the first three months was equally distributed in terms of the age, gender, previous occurrence or existence of atrial fibrillation, previous stroke, previous cardiomyopathy, prior percutaneous coronary intervention (PCI), previous IM and CABG, arterial hypertension, hyperlipidemia, smoking, and positive family history for cardiovascular diseases (Table 1).

Coronary artery disease was more frequent in participants with cardiovascular complications during the follow-up period than those without complications (37.9% vs. 16.1%, p=0.025).

Participants with cardiovascular complications had more frequent diabetes mellitus, particularly those requiring insulin therapy (51.7% vs. 24.7%, p=0.012, that is, 34.5% vs. 9.7%, p=0.003).

The concentration of hsCRP was statistically lower in participants who had cardiovascular complications during the follow-up period, and the levels of NT pro-BNP were significantly higher in those participants. The concentrations of hs TnI and creatine kinase (CK-MB) were significantly higher in participants who did not develop complications (Table 1).

The majority of participants with cardiovascular complications had an RCRI score of 2 or 3 (58.6%), and in those without cardiac complications the most frequently RCRI score was 1 (55.9% of participants). There was a statistically significant difference in the RCRI score between participants with or without cardiac complications (p<0.001).

The type of surgical intervention was not significantly different between participants with or without cardiac complications during the follow-up. In both groups the most common surgery was open surgical reconstruction of the internal carotid artery (with cardiac vs. without cardiac complication: 41.4%, vs. 51.6%).

In Table 2 we inserted medications used by patients with and without cardiovascular complications.

Independent predictors of the development of CV complications in the first 3 months were diabetes mellitus (HR 2.490, p=0.014), V-POSSUM- physiology score (HR 1.179, p<0.001), V-POSSUM-morbidity (HR 1.032, p<0.001), V-POSSUM-mortality (HR 1.193, p=0.003), number of days spent in the ICU (HR 1.668, p<0.001), CK-MB (HR 1.006, p=0.001), NT pro-BNP (HR 5.482, p<0.001), and upper quartile of NT pro-BNP (HR 9.661, p<0.001) (Table 3).

All combinations of biomarkers and clinical scores have good discriminatory/predictive value for the development of cardiovascular complications during the 3 months after surgery AUC>0.8. By comparing the different combinations of clinical scores and biomarkers, we found that RCRI in combination with hsTnI has excellent discriminatory ability for the development of CV complications (AUC 0.909, p<0.001). By adding the V-POSSUM to the RCRI+hsTnI combination we established an even better predictor of CV complications (AUC 0.955, p<0.01). And by adding the NT pro-BNP to RCRI+hs TnI+V-POSSSUM we achieved the best predictive score for CV complications in our participants (AUC 0.963, p<0.001) over a 3-month period. This model (RCRI+NT pro-BNP+V-POSSUM+hsTnI) had significantly better discriminative ability compared to all others combinations (Table 4).

In addition, we calculated the net reclassification index (NRI) and the integrated discrimination index (IDI) to measure the quality of improvement of the correct reclassification with the addition of different parameters to the model.

**Figure 1:**
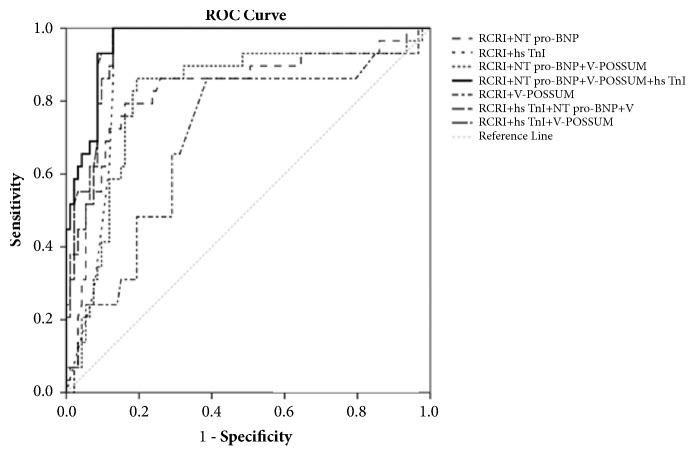
RCRI: Revised Cardiac Risk Index; NT pro-BNP: N-terminal pro b-type natriuretic peptide; hs TnI: high-sensitivity troponin I; V-POSSUM: Vascular Physiological and Operative Severity Score to enumerate the mortality and morbidity; ROC: Receiver Operating Characteristic.

As shown in Table 5, both the NRI and the IDI were significantly improved by the addition of NT pro-BNP to the RCRI (NRI 1.222, p<0.001, IDI 0.229, and p=0.002), and further improvement was noted by adding hs TnI and V-POSSUM (NRI 0.778, p=0.003, IDI 0.289, and p=0.002). Namely, the best predictive tool was the combination of NT pro-BNP, V-POSSUM, and hs TnI with RCRI (NRI 0.783, p=0.003, IDI 0.290, and p=0.001) (Figure 1).

## 4. Discussion

Patients who are admitted for major vascular surgery are at very high risk of postoperative complications due to numerous risk factors such as coronary artery disease, diabetes mellitus, and smoking which accompanies vascular diseases [18]. Accordingly, in our study cardiac complications were more frequent in participants with established coronary artery disease and in those with diabetes mellitus, particularly in those with insulin dependent DM.

The main risk factor for postoperative complications in vascular surgical patients is coronary artery disease. In approximately 4% of major vascular procedures acute myocardial infarction is a complication [19]. In our study group 3.4% participants had AMI as a cardiac complication. Roughly 230 million patients worldwide are subjected to major surgical intervention during a single year, and the incidence of perioperative MI is around 1%. Early mortality after perioperative MI is found within a range of 3.5%-25%. Approximately 80% of perioperative MI is asymptomatic and 60% to 100% has no ST segment elevation (NSTEMI). Often it is very hard to establish a diagnosis and those patients have a poor prognosis [20]. It is very important to detect an adequate rise in troponins or CK-MB to determine the diagnosis. These enzymes are localized in the cytoplasm of cardiomyocytes and are released into circulation in response to irreversible cellular injury and subsequent necrosis. Troponins increase after major vascular surgery, but this is not always a sign of MI. Also, increased levels of hsTnI without myocardial infarction are associated with a poor prognosis. Routine monitoring of these biomarkers has been proposed, with the goal of stratifying the risk of cardiac complications [19].

After identifying high risk, adequate therapy should be immediately implemented to decrease possible postoperative morbidity and mortality [21].

Guidelines recommend the use of clinical risk scores to predict postoperative cardiac events since they are associated with serious morbidity and mortality, as well as health care costs. There is no specific proposed score for patients undergoing arterial vascular surgery [22]. The Revised Cardiac Risk Index (RCRI) was created to predict major cardiac complications after noncardiac surgery [21]. This index takes into account the following: high-risk surgical procedures, history of ischemic heart disease, history of congestive heart failure, history of cerebrovascular disease, DM requiring insulin treatment, and preoperative serum creatinine of >2.0 mg/dL (177 *μ*mol/L). The rates of major cardiac complications with 0, 1, 2, or ≥3 of these factors were 0.4%, 0.9%, 7%, and 11%, respectively. Patients with 3 or more points are marked as high risk and those with 1 or 2 points are considered as being at intermediate risk. The ACC/AHA committee reconsidered the intermediate risk group with 5 risk factors from the original Lee score and ruled out of the type of surgery that is implemented in the other parts of the treatment scheme [20]. The Revised Cardiac Risk Index is in the guidelines published by the European Society of Cardiology, American College of Cardiology, and American Heart Association. It is a clinical tool for perioperative risk stratification in patients undergoing noncardiac surgery [23]. In our study this score was an independent predictor of cardiac complications during a 3-month period.

Gualandro et al. reported that the RCRI and VSG-RCI scores (Vascular Study Group of the New England Cardiac Risk Index) have low precision and low predictive power for the risk of major perioperative cardiac events in vascular surgical patients. Adding preoperative anemia to the RCRI score slightly increases the predictive power [21]. High-sensitivity TnI in combination with RCRI has been shown to be an excellent predictor of early and late mortality in vascular surgery (elective or urgent) patients. There are suggestions that biomarkers should be combined with more suitable stratification tools such as VSG-CRI to obtain maximum predictive power [24]. In practice it is often difficult to select an adequate score for different types of surgery and individual patients. Sometimes, we need to add biomarkers or diagnostic procedures to clinical scores. Evaluation of the functional and exercise capacity is highly recommended by the American Heart Society. In routine practice this is roughly estimated by assessing the daily activities performed by a patient. Generally, patients who can perform exercise at a level above 5 METs (metabolic equivalents) have a low risk of cardiac complications after major surgery [21].

Increased concentrations of hs CRP and NT pro-BNP are markers of future cardiac events. They reflect different pathways of inducing myocardial ischemia postoperatively. Namely, hs-CRP is an inflammatory marker and marker of atherosclerosis. Interestingly, high levels of this biomarker are associated with a worse prognosis independent of any previous manifestations of coronary artery disease. NT pro-BNP is a neurohormone produced by cardiomyocytes as a response to volume and pressure overload. Goei et al. showed that increased concentrations at the baseline (before surgery) of hs-CRP and NT pro-BNP are associated with a 7-fold higher risk of complications in patients who underwent major noncardiac vascular surgery [14]. In our participants low hs CRP levels were associated with increased risk of cardiovascular complications. This could be due to a slow increase in this biomarker or the use of antibiotics [25]. Statins have many mechanisms through which they express beneficial effect perioperatively. Their antithrombotic effects unrelated to cholesterol reduction and anti-inflammatory effects through the downregulation of cytokines are very well known [26]. Our patients with CV complications had less frequent statins in therapy (Table 2).

A high NT pro-BNP value has been shown to indicate a higher risk of cardiac events, even in low-clinical-risk patients who are undergoing vascular surgery. This biomarker had superior predictive power for cardiovascular and all-cause death compared to TnI, CRP, and cystatine C in a population based study in males [27].

In several studies it was noted that heart failure is a powerful marker of perioperative and long-term cardiac prognosis after vascular surgery.

A study by Schouten et al. pointed out that NT pro-BNP has the ability to predict not only perioperative risk but also long-term cardiac risk in patients scheduled for vascular surgery [28].

The difference between BNP and NT pro-BNP concentrations before and after surgery is important. The concentration dynamic indirectly reveals catecholamines peaks during and after surgery, as well as increased coagulability and the risk of prolonged myocardial ischemia. The measurement of NT pro-BNP may be preferred over BNP in patients with preserved renal function because of a longer half-life and a lower susceptibility to being influenced during surgery. Therefore, the postoperative measurement of NT pro-BNP adds additional prognostic information to that which was obtained at the baseline [29].

Biccard et al. showed that the measurement of preoperative BNP concentrations adds additional prognostic power to the RCRI score. Actually, BNP levels reclassify the intermediate risk of RCRI into high and low. Patients at high risk need more diagnostic procedures or therapy, while low risk patients can undergo surgery [30].

Biccard et al. also showed that, in major vascular surgery, besides BNP, troponins have prognostic importance and can be added to the widely accepted RCRI, even though they are inferior compared to preoperatively measured BNP in the net reclassification analysis. This could be the consequence of quick BNP secretion as a response to increased volume or pressure load, or myocardial ischemia. Troponins are secreted later, after myocardial injury or necrosis. A few studies show that troponins peak on the third day after periprocedural MI. There is a weak correlation between before-surgery troponins and postsurgery cardiac complications [31]. Namely, our patients with cardiac complications had significantly lower concentrations of hsTnI. However, in our participants, hs TnI, but not NT pro-BNP, increased the discriminative power of the RCRI score. This could be due to the fact that natriuretic peptides are unstable in rapid hemodynamic/fluid changes and have rapid dynamic clearance [29]. We can only hypothesize that since troponins levels are influenced by hemodynamic status, rhythmic stability, use of betablockers, statins, ACE inhibitors etc., and they should be used together with other variables in the preoperative risk assessment (but not in myocardial ischemia or necrosis settings). Ideal predictive biomarker does not exist. Namely, compared to high-sensitivity troponin T, midregional proadrenomedulin was more effective as a predictor of perioperative cardiovascular complications [32].

The Portsmouth Physiological and Operative Severity Score for the enumeration of the mortality and morbidity (P-POSSUM) score includes 18 markers (duration of surgery, total blood loss, etc.) with the aim of predicting mortality and morbidity when taking into consideration parameters both during the procedure itself and immediately after. It is complex but it shows good predictive power in heterogeneous groups of surgical patients [20]. The POSSUM score has both physiological and operative components. It predicts prognosis before surgery if we use only the physiological component.

Later, P-POSSUM was designed for general surgical patients since POSSUM had low predictive power. It overestimated the risk of mortality. However, both POSSUM and P-POSSUM showed good performance in vascular surgical patients. Their scores were modified later to improve risk stratification performance in vascular surgery, and V-POSSUM was constructed by Vascular Surgical Society for Great Britain and Ireland (VSSGBI). The physiology-only P-POSSUM model has the lowest accuracy of all mentioned scores [29]. In our study physiology V-POSSUM, morbidity v-POSSUM, and mortality-V-POSSUM were independent predictors for cardiac complications.

By adding the V-POSSUM score we increased the discriminatory power of RCRI+hs TnI, and by adding NT pro-BNP we achieved the best predictor for cardiac complications during hospitalization and for a 3-month period after hospital discharge for elective vascular surgical patients. Adding one clinical score and two biomarkers (determined preoperatively) to the RCRI we obtained a new score with the highest NRI and IDI indexes for a 90-day prognosis regarding MACE. It is widely acknowledged that the proposed clinical scores for major vascular surgery are far from perfect and that we need to use other tools such as biomarkers in clinical practice to achieve adequate risk stratification. Trends over the past 10 years are the addition of biomarkers to clinical scores. The RCRI score combined with hsTnI is only a powerful predictive tool for the development of major adverse cardiac events for a 3-month period (including a hospital stay). In our study we showed for the first time, to the best of our knowledge, that a combination of RCRI and V-POSSUM scores with preoperatively measured hs TnI and NT pro-BNP is the better predictive model, with excellent specificity and sensitivity for prediction of 3-month cardiac complications in major vascular surgery.

## 5. Limitations of the Study

This was a single-center study. Only participants who had undergone formal preoperative cardiovascular consultation were included. The presence of preoperative infection and the use of antibiotics, which might affect the level of CRP or the severity of perioperative systemic inflammation, were not used as exclusion or stratification criteria. Patients undergoing urgent surgery were not included among those with a ruptured aortic abdominal aneurysm. They represent a special vulnerable group and should be separately assessed for operative risk. Because of the small number of complications, the result of our research should be interpreted with reservation. Further research on a large number of subjects in multicentre settings and with a greater number of complications is necessary.

## Figures and Tables

**Table 1:** Demographic and clinical characteristics in comparison to the occurrence of cardiovascular complications during the follow-up.

Parameter	With CV compl.		Without CV compl.		**p** ^1^
	Number	%	Number	%	
**Age †**	68.55 ± 6.03		66.56 ± 6.59		0.135^2^
**Gender**					
**Male**	26	89.7	68	73.1	0.110
**Female**	3	10.3	25	26.9	
**Atrial fibrillation**	3	10.3	3	3.2	0.291
**Previous stroke**	5	17.2	27	29.0	0.308
**Coronary artery disease**	11	37.9	15	16.1	0.025
**Cardiomyopathy**	6	20.7	6	6.5	0.059
**Prior PCI**	2	6.9	5	5.4	1.000
**Previous MI**	8	27.6	13	14.0	0.158
**Previous CABG**	1	3.4	1	1.1	0.697
**Arterial hypertension**	25	86.2	79	84.9	1.000
**Diabetes mellitus (DM)**	15	51.7	23	24.7	0.012
**Insulin dependent DM**	10	34.5	9	9.7	0.003
**Hyperlipidemia**	7	24.1	24	25.8	1.000
**Smoking**	13	44.8	36	38.7	0.712
**Positive family history **	14	48.3	35	37.6	0.422
**Type of surgery**					
** Aortic bifemoral bypass **	2	6.9	6	6.5	0.363^1^
** Femoropopliteal bypass **	4	13.8	19	20.4	
** Carotid endarterectomy**	12	41.4	48	51.6	
** Aortoiliac bypass **	11	37.9	20	21.5	
**Number of days in ICU**	2.86 ± 1.53		1.69 ± 1.23		<0.001^3^
**BMI**	25.76 ± 1.96		25.89 ± 2.87		0.785^2^
**hs CRP**	2.69 ± 3.22		3.46 ± 17.29		0.001^3^
**NT pro-BNP**	862.59 ± 13505.93		256.08 ± 575.12		<0.001^3^
**CK-MB**	54.14 ± 87.81		18.63 ± 17.78		<0.001^3^
**hsTnI**	0.00 ± 0.00		0.003 ± 0.004		<0.001^3^

† Arithmetic median ± standard deviation, ^1^Hi-square test, ^2^T test, ^3^Mann-Whitney test. *Abbreviations.* PCI: percutaneous coronary intervention; CABG: coronary artery bypass graft; MI: myocardial infarction; DM: diabetes mellitus; ICU: intensive care unit; BMI: body mass index; hsCRP: high sensitivity C-reactive protein; NT pro-BNP: N-terminal brain natriuretic peptide; CK-MB: creatine kinase MB isoform; hsTnI: high sensitivity troponin I.

**Table 2:** Drugs in patients with and without CV complications.

Therapy	with CV complications		Without CV complications		p^1^
	Broj	%	Broj	%	
Beta blocker	23	79.3	67	72.0	0.593
ACE inhibitors	24	82.8	62	66.7	0.154
Calcium channel blocker	12	41.4	18	19.8	0.036
Antiaggregation therapy	24	82.8	55	59.1	0.036
Statins	12	41.4	45	48.4	0.655
Diuretics	3	10.3	6	6.5	0.769
Long acting nitrates	2	6.9	2	2.2	0.512

^1^Chi-square test.

**Table 3:** Predictors of cardiovascular complications: Cox univariate regression analysis.

**Predictor**	**OR**	**95**%** CI**	**p**
**Age**	1.039	0.980-1.101	0.201
**Male gender**	2.811	0.851-9.290	0.090
**Atrial fibrillation**	2.388	0.721-7.906	0.154
**Arterial hypertension**	1.052	0.366-3.024	0.924
**Diabetes mellitus**	2.490	1.201-5.160	0.014
**Hyperlipidemia**	0.918	0.392-2.150	0.845
**Smoking**	1.290	0.620-2.682	0.496
**Positive family history for CVD**	1.531	0.739-3.172	0.252
**BMI**	0.988	0.864-1.130	0.864
**V-POSSUM Physiology score**	1.179	1.098-1.266	<0.001
**V-POSSUM Operative severity score**	1.020	0.882-1.180	0.789
**V-POSSUM Morbidity**	1.032	1.015-1.050	<0.001
**V-POSSUM Mortality**	1.193	1.062-1.339	0.003
**RCRI**	1.412	0.931-2.139	0.104
**Number of days in the ICU**	1.668	1.318-2.111	<0.001
**Hs CRP**	0.997	0.971-1.024	0.833
**CK-MB**	1.006	1.002-1.009	0.001
**Hs TnI**	5.482	2.953-10.176	<0.001
**logNT pro-BNP**			
**upper quartile ≥365**	9.661	4.386-21.332	<0.001

OR: odds ratio, 95% CI: 95% confidential interval. *Abbreviations.* CVD: cardiovascular disease; BMI: Body Mass Index; V-POSSUM: Vascular Physiological and Operative Severity Score for the enumeration of Mortality and Morbidity; RCRI: Revised Cardiac Risk Index; ICU: Intensive Care Unit; hsCRP: high sensitivity C-reactive protein; NT pro-BNP: N-terminal brain natriuretic peptide; CK-MB: creatine kinase MB isoform; hsTnI: high sensitivity troponin I.

**Table 4:** **Discriminative ability **of different biomarker combinations.

**Model**	**Combinations of biomarkers** **and clinical scores**	**AUC**	**SE**	**95**%** CI**	**Comparison vs Model 7**	
					ΔAUC	p-value^1^
**1**	RCRI+V-POSSUM	0.702	0.057	0.590-0,814	0,261	<0,001
**2**	RCRI+NT pro-BNP+V-POSSUM	0.815	0.049	0.718-0,912	0,148	0,003
**3**	RCRI+NT pro-BNP	0.830	0.049	0.733-0,927	0.133	0.010
**4**	RCRI+hsTnI	0.909	0.027	0.856-0,961	0.055	0.003
**5**	RCRI+hsTnI+NT pro-BNP	0.945	0.019	0.907-0,982	0,017	0,035
**6**	RCRI+hsTnI+V-POSSUM	0.955	0.017	0.922-0,988	0.008	0.044
**7**	RCRI+NT pro-BNP+V-POSSUM+hsTnI	0.963	0.014	0.935-0,992	-	

*Abbreviations. *V-POSSUM: Vascular Physiological and Operative Severity Score for the enumeration of Mortality and Morbidity; RCRI: Revised Cardiac Risk Index; hsCRP: high sensitivity C-reactive protein; NT pro-BNP: N-terminal brain natriuretic peptide; hsTnI: high sensitivity troponin I, Se: sensitivity, AUC: area under curve, and ^1^DeLong test.

**Table 5:** NRI and IDI for combined markers.

Combination of biomarkers	NRI	95% CI	p	IDI	95% CI	p
**RCRI**	referent group					
**RCRI+NT pro-BNP**	1.222	0.951-1.493	<0.001	0.229	0.115-0.463	0.002
**RCRI+hs TnI**	0.222	-0.795-1.239	0.668	0.048	-0.143-0.239	0.690
**RCRI+VPOSSUM**	0.222	-0.795-1.239	0.668	0.032	-0.132-0.203	0.714
**RCRI+NT pro-BNP+V-POSSUM**	-0.278	-1.169-0.613	0.541	0.013	-0.102-0.127	0.827
**RCRI+ hs TnI +NT pro-BNP**	-0.222	-1.328-0.884	0.693	0.031	-0.143-0.206	0.724
**RCRI+ hs TnI +V-POSSUM**	0.778	0.264-1.291	0.003	0.289	0.115-0.463	0.002
**RCRI+NT pro-BNP+V-POSSUM+ hs TnI**	0.783	0.254-1.301	0.003	0.290	0.117-0.470	0.001

NRI: net reclassification index, IDI: integrated discrimination index. *Abbreviations.* CVD: cardiovascular disease; BMI: Body Mass Index; V-POSSUM: Vascular Physiological and Operative Severity Score for the enumeration of Mortality and Morbidity; RCRI: Revised Cardiac Risk Index; ICU: Intensive Care Unit; hsCRP: high sensitivity C-reactive protein; NT pro-BNP: N-terminal brain natriuretic peptide; CK-MB: creatine kinase MB isoform; hsTnI: high sensitivity troponin I.

## Data Availability

The data used to support the findings of this study are available from the corresponding author upon request.
